# A 3D bioengineered human liver for the study of acute and chronic drug-induced hepatotoxicity and fibrosis

**DOI:** 10.3389/fbioe.2026.1798323

**Published:** 2026-05-11

**Authors:** Ainhoa Ferret-Miñana, Estefanía Alcaraz, Raquel Horrillo, Javier Ramón-Azcón, Francesco De Chiara

**Affiliations:** 1 Biosensors for Bioengineering Group, Institute for Bioengineering of Catalonia (IBEC), Barcelona, Spain; 2 Universitat de Barcelona (UB), Barcelona, Spain; 3 Scientific Innovation Office, Grifols, Barcelona, Spain; 4 ICREA-Institució Catalana de Recerca i Estudis Avançats, Barcelona, Spain

**Keywords:** 3d liver model, acute liver injury, chronic liver injury, drug-induced hepatotoxicity, hepatic fibrosis, hepatic stellate cells, liver disease modeling

## Abstract

The liver is frequently exposed to acute and chronic insults that disrupt its function, leading to inflammation, fibrosis, and serious conditions like cirrhosis and hepatocellular carcinoma. Fibrosis, driven by the activation of hepatic stellate cells is a key feature of chronic liver damage. Traditional 2D liver models face limitations in maintaining liver functions and accurately replicating chronic conditions like fibrosis. In response to these needs, 3D models have emerged as more physiologically relevant platforms for studying liver disease and drug testing. However, most existing 3D models still lack chronic injury features or immune elements. In this study, we developed a 3D human-liver model to investigate both acute and chronic drug-induced liver injury. The model consists of encapsulated human hepatocytes (HepaRG) and hepatic stellate cells (LX-2) within a gelatin methacryloyl (GelMA) and carboxymethyl cellulose methacrylate matrix. Over a 30-day culture period, the 3D liver constructs maintained physiologically relevant functions, including albumin secretion and cytochrome P450 activity, which are lost rapidly in conventional 2D models. The model enabled reliable simulation of low-level, long-term, and high-dose, short-term damage by administering lipopolysaccharide and Paracetamol. Chronic liver injury was characterized by progressive fibrosis, elevated expression of COL1A1, and persistent hyperammonemia. In contrast, acute models showed significant but transient injury. Moreover, dexamethasone treatment successfully reversed fibrotic markers and restored hepatocyte functionality, indicating the model’s predictive power for evaluating potential therapies. Incorporating human monocytes (THP-1) allowed investigation of the immune response and macrophage activation, which are critical contributors to liver disease pathogenesis. Overall, this 3D liver model offers a physiologically relevant and versatile platform for studying complex multi-cellular interactions under acute and chronic injury. It has broad implications for drug safety screening, disease modeling, and personalized therapy. The tri-culture design extends the model’s capacity to elucidate immune-driven hepatic pathology.

## Introduction

Liver disease remains a critical global health issue, accounting for over two million deaths annually. Due to the liver’s central role in metabolism, detoxification, and regulating various physiological processes, even mild hepatic dysfunction can exert profound systemic effects. Liver diseases stem from diverse causes, including viral infections, alcohol intake, high-fat/high-sugar diets, and drug-induced liver injury (DILI) arising from certain pharmaceuticals or toxins (Wang R, 2023). These insults may manifest in two primary forms: acute and chronic. Acute liver failure (ALF) typically develops rapidly in individuals without prior liver disease, frequently triggered by DILI or viral infections (Sowa JP, 2016). In contrast, chronic liver disease (CLD) arises from sustained injury and inflammation, eventually leading to fibrosis and cirrhosis (Taru V, 2024). Cirrhosis, the advanced stage of CLD, involves excessive tissue scarring, abnormal regenerative nodules, and compromised liver function—culminating in liver failure or hepatocellular carcinoma if left untreated (Somnay K, 2024).

A pivotal element in acute and chronic liver damage is hepatic stellate cells (HSCs) activation. In response to hepatocyte injury, HSCs transition into activated myofibroblasts, overproducing extracellular matrix components and driving fibrosis (Akkız H, 2024). This process is governed by complex signals between different hepatic cell populations. Alongside HSCs, immune cells—particularly monocytes and macrophages—play a central part in orchestrating liver injury. Following damage, monocytes are recruited to the liver, differentiating into macrophages that secrete pro-inflammatory cytokines and chemokines (e.g., TNF-alpha, IL-1beta, TGF-beta) (Xu J, 2014). These mediators propagate hepatocyte injury and further activate HSCs, exacerbating fibrosis.

Traditional two-dimensional (2D) *in vitro* models struggle to replicate the liver’s complex microenvironment and sustain liver-specific functions. Moreover, HSCs cultured on rigid 2D surfaces often undergo spontaneous activation due to the high stiffness of these materials, deviating from physiologically relevant behavior and limiting the study of fibrotic mechanisms (Brovold, 2020). Thus, more refined and physiologically relevant models are needed for investigating liver disease and for preclinical drug testing.

Three-dimensional (3D) *in vitro* liver models address these limitations by better mimicking liver architecture, cell-cell interactions, and the extracellular matrix (ECM). Co-culture systems incorporating parenchymal hepatocytes with non-parenchymal cells—including HSCs, liver sinusoidal endothelial cells, Kupffer cells, and immune cells—enable deeper insights into hepatic homeostasis, intercellular communication, and disease pathogenesis.

3D liver models also improve the prediction of drug-induced liver injury (DILI), which accounts for ∼33% of post-market withdrawals. Unlike 2D cultures, 3D spheroids and microtissues maintain stable expression of phase I/II enzymes (e.g., CYP3A4, 2D6), transporters (BSEP, MRP2), and stress/death pathways, enhancing sensitivity (80%–90%) and specificity (85%–95%) for diverse hepatotoxic mechanisms (Zhou et al., 2019; Yang et al., 2023). Proctor et al. (Proctor et al., 2017) showed that liver microtissues combining hepatocytes with HSCs, Kupffer, and endothelial cells outperformed 2D systems (87% sensitivity, 92% specificity) and better reflected clinical DILI data. Bell et al. (Bell CC D. A.-Y.-S., 2018) demonstrated that PHH spheroids maintain ADME protein expression, CYP activity, and proteome stability over 14 days, surpassing 2D monolayers and sandwich cultures for repeated-dose and mechanistic studies.

Long-term, multicellular 3D models further enable mechanistic insights. They preserve hepatocyte function 5-10x longer than 2D cultures, reveal cumulative mitochondrial toxicities [179], and amplify fibrogenic responses via stellate and immune co-cultures (Sun et al., 2024). High-throughput microphysiological systems allow quantitative assessment of fibrosis progression alongside acute DILI, supporting early drug development (Schmidt and Suter-Dick, 2025).

However, most current 3D liver models do not simulate long-term fibrotic progression or include immune components necessary to recapitulate chronic inflammation. In this study, we developed a human-relevant 3D liver model by encapsulating human hepatocytes (HepaRG) and HSCs (LX-2) in a gelatin methacryloyl (GelMA) and carboxymethyl cellulose methacrylate (CMCMA) matrix, co-cultured with monocytes (THP-1). Our work proceeded as follows:

1. Establishing a Long-Term 3D Liver Model: We encapsulated HepaRG and LX-2 cells in GelMA-CMCMA hydrogels for up to 30 days under serum-free conditions, investigating their viability, phenotypic stability, and hepatic functionality over this extended period.
2. Recapitulating Chronic and Acute Liver Damage: We induced chronic and acute liver injury by exposing tissues to lipopolysaccharide (LPS) and Paracetamol at different concentrations and durations. Chronic damage resulted from lower-level, prolonged exposure, whereas acute damage was induced by higher concentrations over a shorter timeframe.
3. Validating Model Responses with Dexamethasone: To assess the model’s predictive capability for drug efficacy, we treated the injured liver tissues with dexamethasone—an anti-inflammatory and antifibrotic glucocorticoid—and quantified improvements in hepatocyte function and fibrotic markers.
4. Incorporating Immune Cells for a Tri-Culture System: We integrated THP-1 monocytes to evaluate how immune cell recruitment and activation contribute to liver injury. By placing 3D liver tissues and monocytes in transwell co-culture, we observed inflammatory signaling events, which were especially relevant for understanding the synergistic role of macrophages and HSCs in driving chronic and acute hepatic damage.

This study is designed to provide a versatile and physiologically relevant platform for investigating liver pathophysiology, including chronic and acute injury mechanisms, fibrotic progression, and immune-mediated responses. By combining hepatocytes, HSCs, and monocytes within a durable 3D hydrogel, the model aims to capture the complex multicellular interactions that drive liver disease, while offering a controlled system for evaluating pharmacological interventions. Overall, this approach establishes a human-relevant framework for studying liver function, intercellular crosstalk, and therapeutic modulation under long-term culture conditions.

## Results

### Healthy liver: sustained hepatocyte viability and organization in the 3D liver model over 30 days

**FIGURE 1:**
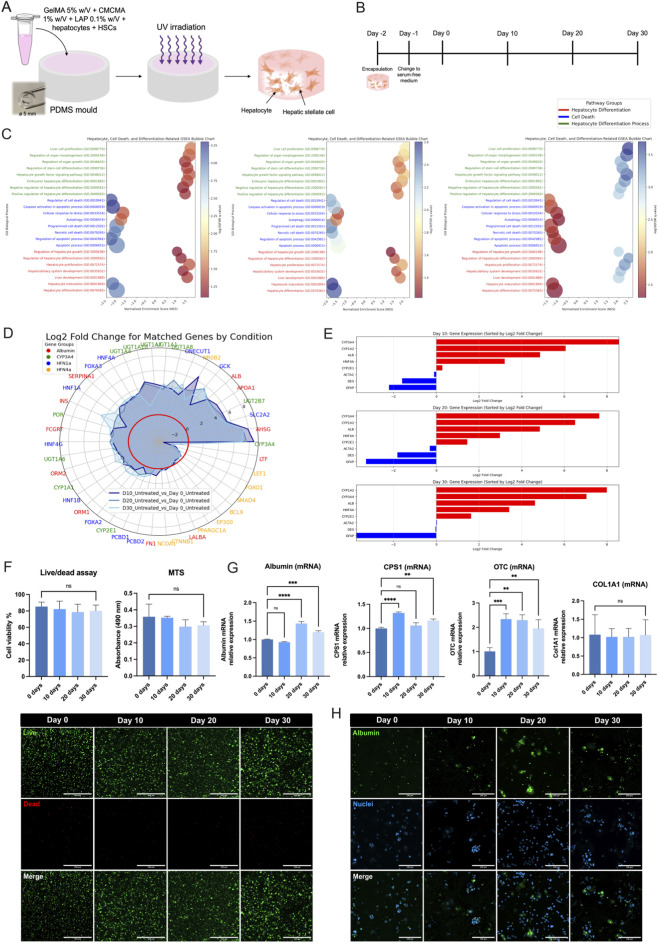
Fabrication and characterization of the 3D liver model cultured for 30 days. **(A,B)** Schematic representation of the fabrication process of the 3D liver model. Hepatocytes (HepaRG) and hepatic stellate cells (LX-2) are encapsulated within a GelMA-CMCMA hydrogel matrix. Cells were encapsulated on Day −2, transferred to serum-free medium (SFM) on Day −1, and challenged with treatments starting at Day 0. Samples were collected at Days 0, 10, 20, and 30 for analysis. **(C)** Gene Set Enrichment Analysis (GSEA) of biological processes related to hepatocyte differentiation (green), cell death (blue), and hepatocyte maturation (red) over time. The bubble plots show normalized enrichment scores (NES) and adjusted p-values across Days 10, 20, and 30 (left to right). **(D)** Radar chart showing the log2 fold change of selected hepatocyte-related genes grouped by interactors (string database) of Albumin, CYP3A4, HNF1a, HNF4a. **(E)** Bar graphs of gene expression changes (log2 fold change) for selected genes at Days 10, 20, and 30. Red bars represent upregulated genes, while blue bars represent downregulated genes. **(F)** Live/Dead and MTS assays. The Live/Dead assay shows consistent cell viability across time points, while the MTS assay reveals stable metabolic activity. **(G)** mRNA expression levels measured by qPCR of hepatocyte markers (Albumin, CPS1, OTC) and the fibrosis marker Col1α1 across time points. **(H)** Immunofluorescence imaging of albumin (green), nuclei (blue), and merged images over 30 days. The statistical significance is so represented: *= 0.05; **= 0.01; ***= 0.001; ****= 0.0001 in at least three independent experiments. These results validate the establishment and characterization of the 3D liver model, providing insights into hepatocyte function, gene expression dynamics, and metabolic responses over time. The model demonstrates its suitability for studying chronic liver injury and associated cellular processes.

We employed a hydrogel moulding technique to fabricate human three-dimensional (3D) liver tissues (Figure 1A). Polydimethylsiloxane (PDMS) moulds, each featuring a 5 mm inner diameter and 8 mm outer diameter ring, were used to encapsulate immortalized human hepatocytes (HepaRG; Heps) and hepatic stellate cells (LX-2; HSCs) at a 2:1 ratio (Heps:HSCs) within a blend of GelMA and CMCMA. The hydrogels were then crosslinked under ultraviolet (UV) light to form stable 3D liver tissues that replicated key features of the liver microenvironment. On Day 1, the tissues were transferred from a growth medium to a serum-free differentiation medium (SFM). Cultures were maintained for up to 30 days, with media changes every 2–3 days. Sample analyses were performed on Days 0, 10, 20, and 30 to evaluate long-term viability and functionality (Figure 1B). This approach builds upon our previous work in developing a 3D bioengineered liver model to study hepatic steatosis in murine hepatocytes under high-fat conditions (De Chiara F, 2022). However, to investigate more advanced stages of liver disease, such as fibrosis and inflammation, it was necessary to include non-parenchymal cells (HSCs) and immune cells (monocytes).

### Gene set enrichment analysis: hepatocyte differentiation, cell death, and maturation

To elucidate the biological processes underlying hepatocyte differentiation, cell death, and hepatocyte maturation in the 3D liver model, we performed gene set enrichment analysis (GSEA) on transcriptomic data from both Heps and HSCs. The goal was to characterize the temporal evolution of critical pathways. Hepatocyte differentiation (green) consistently exhibited positive normalized enrichment scores (NES), which increased from 1.5 (FDR = 0.05) at Day 10 to 2.5 (FDR <0.001) at Day 30, indicating a robust and progressive enhancement of the hepatocyte phenotype. Conversely, Cell death pathways (blue) exhibited negative NES values throughout, indicating the suppression of apoptotic and necrotic events. Notably, the significance of these pathways decreased from Day 10 to Day 30, moving from NES -2.0, FDR = 0.001 at Day 10 to NES -1.0, FDR = 0.05 at Day 3, reflecting a decline in injury-induced cell death over time. Hepatocyte maturation processes (red) demonstrated a dynamic but statistically more modest profile; these pathways reached significance later in the culture (e.g., FDR improved from 0.08 at Day 10 to 0.001 at Day 30), with NES values (max NES 2.0) that remained lower than those observed for the differentiation program (Figure 1C). These statistical benchmarks support the interpretation of a steady differentiation phase followed by a more gradual maturation trajectory.

### Time-dependent changes in hepatocyte function

Next, we investigated time-dependent changes in hepatocyte function by examining the expression of hepatocyte-specific markers, including Albumin, CYP3A4, HNF1A, and HNF4A, as well as their interacting partners identified in the STRING database (Figure 1D). Log_2_ fold-change values were calculated on Days 10, 20, and 30 relative to Day 0. CYP3A4, NR0B2, and FN1 were downregulated over time, suggesting reduced xenobiotic metabolism, transcriptional regulation, and extracellular matrix (ECM) remodeling, respectively. In contrast, GCK, INS, CYP1A1, ORM1, and CYP2E1 displayed upregulation, underscoring improved glucose handling, insulin signaling, acute-phase responses, and phase I detoxification. Collectively, these changes confirm that hepatocytes adapt over time to the 3D culture, exhibiting a maturation of liver-specific functions.

Parallel analyses revealed the downregulation of HSC biomarkers, indicating a quiescent stellate cell phenotype in the absence of sustained fibrotic stimuli. Specifically, the expression levels of ACTA2 (α-SMA), DES (desmin), and GFAP steadily decreased over time, with ACTA2 and DES log_2_ fold changes approaching zero by Day 30 (Figure 1E). GFAP levels similarly dropped, reinforcing the notion that HSCs transitioned to a quiescent state as the culture progressed.

### Long-term viability and functionality of the 3D liver model

To confirm cell viability in the 3D liver model over a 30-day period, we performed an MTS enzymatic assay and a live/dead visual assessment. Neither method revealed significant differences over the culture period, indicating that metabolic activity and cell viability were well maintained (Figure 1F). CYP3A4 enzymatic activity significantly increased at days 10, 20 and 30 when compared to day 0 (p < 0.0001) (Supplementary Figure S1). We then quantified the expression of key liver-specific genes—Albumin, Ornithine transcarbamylase (OTC), and Carbamoyl phosphate synthetase I (CPS1)—as well as the fibrotic marker collagen type I alpha 1 (Col1α1). Albumin expression increased significantly on Days 20 and 30 (p < 0.0001 and p < 0.001, respectively). CPS1 mRNA increased markedly between Day 10 (p < 0.0001) and continued to rise moderately by Day 30 (p < 0.01). OTC levels also exhibited significant increases from earlier time points to Days 20 and 30 (p < 0.001, p < 0.01, and p < 0.01, respectively). In contrast, Col1α1 remained relatively stable across all time points, showing no significant changes (Figure 1G).

Confocal immunofluorescence imaging corroborated these findings by confirming consistent Albumin expression and intact nuclei (as indicated by DAPI staining) throughout the 30-day culture period. Both hepatocytes and HSCs formed stable clusters within the biomaterial matrix, underscoring the capability of this 3D environment to promote cell-cell interactions and sustained viability (Figure 1H; Supplementary Figure S2).

Overall, these results highlight the robustness of our 3D liver model in supporting hepatocyte-specific functions and organizing both parenchymal and non-parenchymal cells over extended culture periods. The progressive enhancement of hepatocyte metabolic activity, alongside the quiescent state of HSCs, underscores the suitability of this model for long-term studies of liver physiology and disease processes.

#### Chronic liver damage model

**FIGURE 2:**
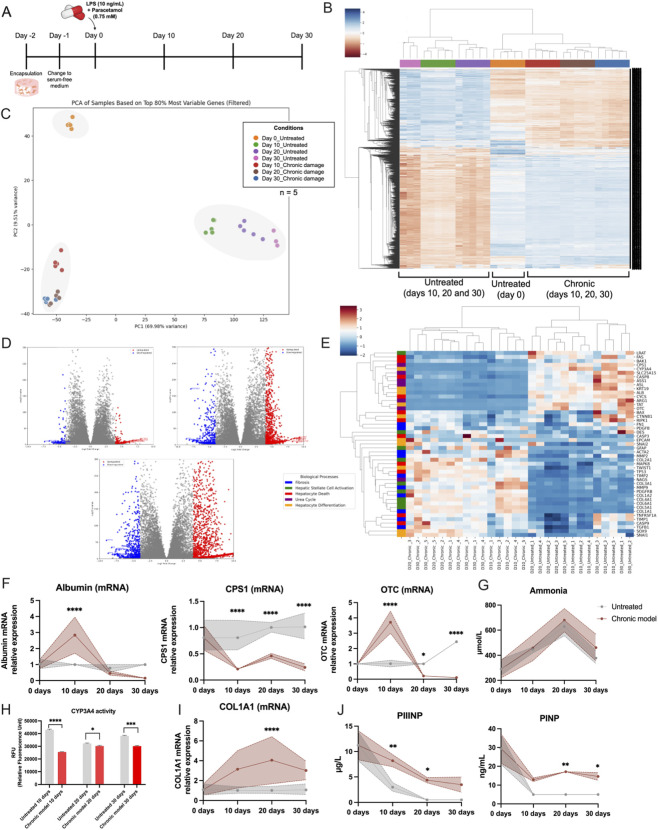
*In vitro* three-dimensional model of chronic liver damage using human hepatocytes and hepatic stellate cells. **(A)** Experimental timeline. Encapsulation of hepatocytes and hepatic stellate cells was performed on Day −2, followed by a transition to serum-free medium (SFM) on Day −1. Chronic liver damage was induced on Day 0 using LPS (10 ng/mL) and Paracetamol (0.75 mM). Samples were collected at Days 10, 20, and 30 for analysis. **(B)** Principal component analysis (PCA) of transcriptomic data based on the top 80% most variable genes (n = 5 per group). **(C)** Heatmap displaying the top 10% most differentially expressed genes across all samples. **(D)** Volcano plots comparing chronic-damage samples with their time-matched untreated controls (Day 10: top-left; Day 20: top-right; Day 30: bottom). Genes with log_2_ fold change ≥1 and adjusted p ≤ 0.001 are coloured red (upregulated); those with log_2_ fold change ≤−1 and adjusted p ≤ 0.001 are coloured blue (downregulated). **(E)** Heatmap of selected biological processes, including fibrosis, hepatic stellate cell activation, hepatocyte death, urea cycle, and hepatocyte differentiation. **(F,G)** mRNA expression levels of hepatocyte-specific markers (Albumin, CPS1, OTC) and ammonia levels in the medium. **(H)** CYP3A4 activity. **(I)** mRNA expression of COL1A1 (fibrotic marker). **(J)** Enzymatic assays for fibrosis-related markers (PIIINP and PINP). The statistical significance is denoted as follows: *= 0.05; **= 0.01; ***= 0.001; ****= 0.0001. These data highlight the metabolic, transcriptional, and fibrotic changes induced by chronic liver damage in the 3D liver model, demonstrating its utility in studying the mechanisms of liver disease.

To assess how our 3D liver model responds to sustained injury, we treated encapsulated hepatocytes (Heps) and hepatic stellate cells (HSCs) with a combination of lipopolysaccharide (LPS, 10 ng/mL) and paracetamol (0.75 mM) on Day 0 (Figure 2A). This dual treatment was designed to simulate chronic liver damage by inducing ongoing hepatocyte injury and inflammation.

## Global transcriptional changes

We performed principal component analysis (PCA) on the top 80% of the most variable genes to explore how gene expression evolved over 30 days under untreated and chronic damage (LPS-Paracetamol) conditions (Figure 2B). Untreated Day 0 samples clustered at the top-left, representing baseline expression profiles. As the culture progressed to Days 10, 20, and 30 (Untreated), the samples shifted toward the middle-right, reflecting transcriptomic changes associated with hepatocyte differentiation. In contrast, samples from the chronic damage group on Days 10, 20, and 30 clustered toward the bottom-left, demonstrating a transcriptional trajectory distinct from the untreated baseline and the normal differentiation pattern.

Hierarchical clustering and heatmap analysis further highlighted these divergent gene expression patterns (Figure 2C). Notably, the five replicates within each condition clustered tightly together, underscoring the high reproducibility and robustness of the experimental setup. This clear separation between untreated and chronic damage samples confirms that LPS-Paracetamol treatment drives a distinct transcriptional response. Furthermore, an upset plot (Supplementary Figure S3) showed that the comparison between Untreated Day 0 and Chronic Day 30 resulted in the highest number of downregulated genes (528), highlighting the substantial impact of prolonged chronic damage on the transcriptome.

### Time-dependent differential expression

Volcano plots comparing chronic damage versus untreated conditions at Days 10, 20, and 30 revealed the dynamic nature of gene expression in response to prolonged injury (Figure 2D). To gain deeper insight into the underlying biological processes, we performed a targeted heatmap analysis of differentially expressed genes (DEGs) associated with fibrosis, HSC activation, hepatocyte death, urea cycle function, and hepatocyte differentiation (Figure 2E). Under chronic damage, fibrotic and HSC activation pathways showed elevated activation z-scores, indicating enhanced fibrotic remodeling. Similarly, hepatocyte death–related genes were significantly upregulated, reflecting increased cell injury. Conversely, genes linked to the urea cycle (Supplementary Figure S4) and hepatocyte differentiation exhibited higher z-scores in untreated samples, suggesting preserved hepatocyte function under baseline conditions but marked impairment in the chronic damage model.

### qPCR validation of hepatic markers

To corroborate the RNA-seq findings, we performed quantitative PCR (qPCR) for key hepatocyte genes—Albumin, CPS1, and OTC—at Days 10, 20, and 30 in untreated and chronic damage conditions (Figure 2F). In the chronic damage model, Albumin initially rose at Day 10 (p < 0.0001) before sharply declining, indicating a transient reactive response to injury. CPS1 remained stable in untreated samples but decreased significantly under chronic damage, most notably at Day 20 (p < 0.0001), indicating impaired urea cycle function. OTC displayed a similar trend to Albumin, with elevated levels at Day 10 in the chronic damage model, followed by a marked drop by Day 20 and persistently low expression thereafter (p < 0.05 and p < 0.001). Although ammonia levels followed similar patterns in untreated versus damaged samples, actual detoxification depends on multiple factors—enzyme activity, substrate availability, and overall metabolic state, not solely on mRNA abundance.

## Functional assays: CYP3A4 activity and fibrosis markers

CYP3A4 activity remained consistently high in untreated samples, with a modest decline between Days 10 and 30 (Figure 2G). In contrast, chronic damage samples displayed significantly lower CYP3A4 activity at all time points, with an acute reduction on Day 10 (p < 0.0001) and only partial recovery by Day 20 (p < 0.05). Activity subsequently declined again by Day 30 (p < 0.01), underscoring sustained impairment of hepatocyte metabolic capacity under chronic injury conditions.

We next evaluated fibrotic progression by measuring Col1α1, Procollagen III N-terminal propeptide (PIIINP) and Procollagen I N-terminal propeptide (PINP) (Figures 2H,I). COL1A1 mRNA levels progressively increased in the chronic damage model through Days 10, 20, and 30 (p < 0.0001 at day 20), reflecting ongoing HSC activation and extracellular matrix deposition. Similarly, PIIINP and PINP protein levels were significantly higher in the chronic damage group than in untreated samples from Day 10 to Day 30 (p < 0.01). IL-6 (p < 0.0001) and MCP-1 (p < 0.05 and (p < 0.001) protein levels were also significantly increased in the chronic damage group than in untreated samples at days 20 and 30 (Supplementary Figure S5). Untreated cultures maintained consistently low levels of all three markers, indicating minimal fibrotic remodelling in the absence of injury stimuli.

Together, these results demonstrate that LPS-Paracetamol treatment triggers a distinct and progressive injury response in our 3D liver model, encompassing the downregulation of key metabolic functions, enhanced fibrotic pathways, and heightened cell death-related processes. The tight clustering of replicates and temporal specificity of these changes underscore the model’s reliability and its potential to capture the complex transcriptional and functional dynamics of chronic liver disease.

## Confocal microscopy of key markers

**FIGURE 3:**
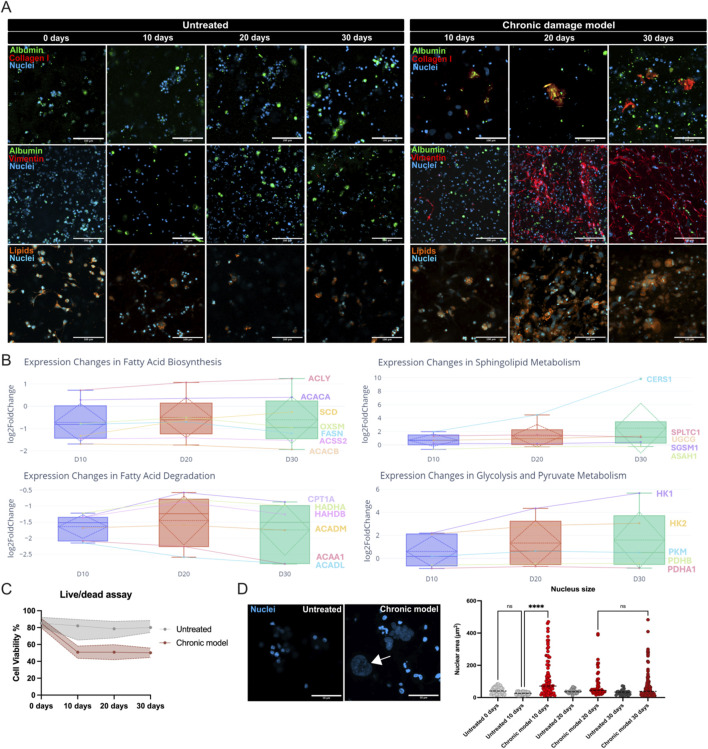
Morphological, transcriptional, and cellular responses in the chronic damage model. **(A)** Immunofluorescence staining comparing untreated and chronic damage conditions across Days 10, 20, and 30. Top row: Albumin (green), Collagen I (red), and nuclei (blue). Scale bar = 100 µm. Middle row: Albumin (green), vimentin (red), and nuclei (blue). Scale bar = 200 µm. Bottom row: Lipid droplets (orange) and nuclei (blue). Scale bar = 100 µm. **(B)** Box plots showing log2 fold changes in genes associated with fatty acid biosynthesis, sphingolipid metabolism, fatty acid degradation, and glycolysis/pyruvate metabolism over Days 10, 20, and 30. **(C)** Live/Dead assay. **(D)** Nuclear morphology at Day 10 in untreated and chronic damage models. Chronic damage results in nuclear enlargement (arrow) compared to untreated samples. **(E)** Quantification of nuclear area reveals significantly larger nuclei in the chronic damage model. The statistical significance is represented as follows: ns = not significant; ****= 0.0001. These results demonstrate the progressive impact of chronic damage on hepatocyte function, lipid metabolism, fibrosis, and nuclear morphology in the 3D liver model, highlighting the model’s utility for studying chronic liver injury and related metabolic dysfunctions.

To visualize the progression of fibrosis, changes in hepatocyte function, and lipid accumulation under chronic damage conditions, we performed confocal microscopy of the 3D liver model at Days 10, 20, and 30 (Figure 3A). DAPI (blue) labelled nuclei, and three panels captured different markers. The top row displays albumin and collagen I. In untreated samples, albumin staining persists across all time points, suggesting sustained hepatocyte functionality. Collagen I expression remains minimal, reflecting the absence of notable fibrogenic activity. In contrast, the chronic damage samples exhibit a gradual increase in collagen I deposition, becoming pronounced by Day 30 and indicating progressive fibrosis; concurrently, albumin staining diminishes at later time points, pointing to impaired hepatocyte function. The middle row shows albumin and vimentin. Untreated samples retain stable albumin expression, while vimentin staining appears faint, signifying minimal HSC activation under normal conditions. However, vimentin becomes markedly elevated in the chronic damage samples, forming dense networks at Days 20 and 30 that reflect robust HSC activation into myofibroblasts (Supplementary Figure S6), and extracellular matrix remodeling. The reduction in albumin staining in these treated samples is consistent with declining hepatocyte functionality within a fibrotic environment. The bottom row depicts lipid staining. Untreated samples show minimal lipid accumulation over time, whereas chronic damage samples exhibit a progressive buildup of large orange droplets, especially by Day 30, indicative of steatosis and disrupted lipid metabolism under conditions of sustained injury.

### Metabolic pathway analysis via RNA-Seq

We cultured the 3D liver tissues in serum-free medium for up to 30 days (n = 5 replicates per condition) and performed RNA-seq to investigate genes involved in fatty acid biosynthesis, fatty acid degradation, sphingolipid metabolism, and glycolysis (adjusted p ≤ 0.05). The fatty acid biosynthesis pathway features upregulated ACACA and ACLY, indicating increased acetyl-CoA generation for lipid synthesis. In contrast, downstream enzymes (FASN, OXSM, ACSS2, SCD) are consistently suppressed, disrupting fatty acid elongation and overall biosynthesis (Figure 3B, top-left panel).

Fatty acid degradation is also impaired, with the downregulation of CPT1A, ACADM, ACADL, HADHA, HADHB, and ACAA1, reflecting compromised mitochondrial beta-oxidation and inadequate fatty acid catabolism. This imbalance suggests lipid accumulation partly results from increased substrate availability (acetyl-CoA) unaccompanied by effective downstream synthesis or oxidation (Figure 3B, bottom-left panel).

Sphingolipid metabolism shows evidence of ceramide accumulation, as SPTLC1 and CERS1 are elevated while ASAH1 is downregulated. Ceramide is implicated in apoptosis, inflammation, and cellular dysfunction, which may contribute to liver injury. However, UGCG is upregulated, which may represent a compensatory mechanism that channels ceramide into less toxic glycosphingolipids (Figure 3B, top-right panel).

Glycolysis is also affected, with HK1, HK2, and PKM upregulated, suggesting heightened glycolytic flux as a compensatory response to injury. The simultaneous suppression of PDHA1 and PDHB impairs the pyruvate dehydrogenase complex, limiting oxidative metabolism and driving a metabolic shift toward less efficient, non-oxidative pathways (Figure 3B, bottom-right panel).

### Cell viability and metabolic activity

Live/Dead assays reveal an approximately 50% decline in cell viability starting at Day 10 in the chronic damage group (Figure 3C), indicating progressive hepatocyte loss and metabolic dysfunction.

### Nuclear morphology changes

To investigate morphological changes, we compared nuclear size between untreated and chronic damage conditions (Figure 3E). The nuclei in untreated samples remain uniform in size, reflecting stable cell health. In contrast, nuclei in the chronic damage model become irregular and enlarged, with a statistically significant increase in nuclear area (p < 0.0001) from Day 10 onward. This morphological hallmark of cellular stress persists through the later time points and is not observed in untreated samples, where nuclear dimensions remain consistent.

These data demonstrate that chronic LPS-Paracetamol exposure triggers a range of responses in the 3D liver model. Fibrosis progresses, evidenced by elevated collagen I and vimentin expression, while hepatocyte function declines, as indicated by diminished albumin staining and increased lipid accumulation. Metabolic pathways undergo significant reprogramming, characterized by impaired fatty acid oxidation, disrupted fatty acid biosynthesis, and ceramide accumulation, alongside a heightened reliance on glycolysis. The overall metabolic activity is further complicated by HSC compensation, which may mask the true extent of hepatocyte damage when evaluating total cellular metabolism. These findings highlight the multifaceted nature of chronic liver injury in the 3D model and underscore its utility for studying complex interactions among fibrosis, metabolic pathways, and cellular stress responses.

### Acute liver damage in the 3D liver model

**FIGURE 4:**
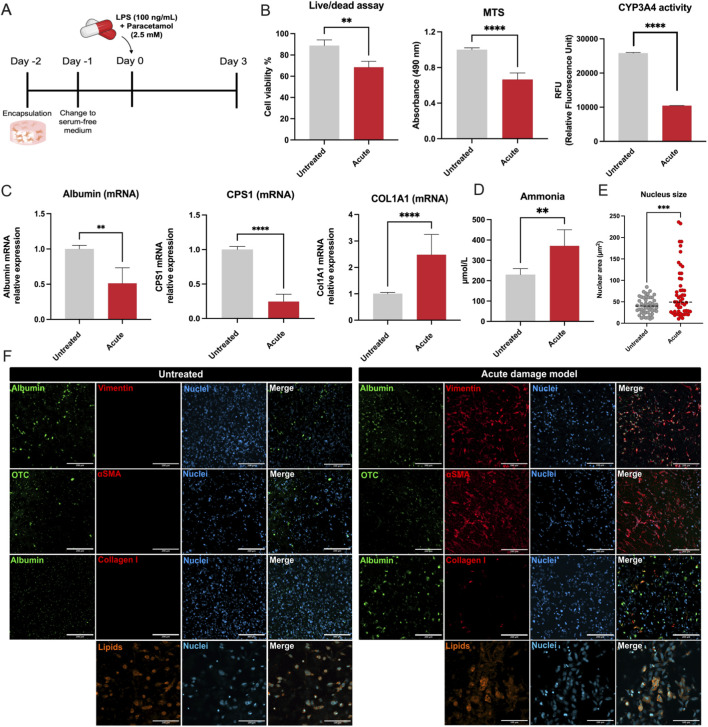
Functional, transcriptional and morphological changes in the acute damage model. **(A)** Experimental timeline. Encapsulation of 3D liver tissues was performed on Day −2, followed by a switch to serum-free medium (SFM) on Day −1. Acute liver damage was induced on Day 0 using LPS (100 ng/mL) and Paracetamol (2.5 mM). Samples were analyzed on Day 3. **(B)** Quantification of cellular viability, metabolic activity, and CYP3A4 activity. **(C)** Hepatocyte-specific and fibrotic markers gene expression. mRNA expression of hepatocyte markers Albumin, CPS1, and COL1A1 measured by qPCR. **(D)** Ammonia levels in the medium. **(E)** Nuclear area. **(F)** Immunofluorescence staining for albumin and OTC in green, fibrosis markers such as vimentin, α-SMA, and Collagen I in red, and nuclei in blue. **(G)** Lipid accumulation. Lipid staining (orange) and nuclear staining (blue). The statistical significance is denoted as follows: *= 0.05; **= 0.01; ***= 0.001; ****= 0.0001. These findings highlight the severe impact of acute liver damage on hepatocyte functionality, metabolic activity, and structural integrity, as well as the activation of fibrogenic responses and dysregulation of lipid metabolism. The 3D liver model effectively recapitulates key aspects of acute liver injury.

To investigate acute liver damage, we transitioned the culture from growth medium to differentiation medium (serum-free medium, SFM) 1 day after cell encapsulation. Two days later, we applied a high-concentration combination of LPS (100 ng/mL) and Paracetamol (2.5 mM), exceeding the concentrations used in the chronic model, to induce acute injury *in vitro*. On Day 3 following treatment, we analyzed the liver tissues to assess the impact of this acute insult (Figure 4A).

Both the Live/Dead and MTS assays showed comparable reductions in viability and metabolic activity, each decreasing by a similar magnitude relative to untreated controls (Live/Dead: *p* < 0.01; MTS: *p* < 0.0001). These findings suggest that hypermetabolic activity, possibly from activated HSCs, obscures the actual loss of hepatocyte viability. Acute damage also resulted in a substantial increase in nuclear size, indicated by a broader distribution and higher mean nuclear area (p < 0.001) (Figure 4B). CYP3A4 enzyme activity dropped markedly compared to untreated samples (p < 0.0001) (Figure 4C). In parallel, mRNA levels of Albumin and CPS1 fell sharply, whereas COL1A1 mRNA rose significantly (p < 0.0001, p < 0.0001, and p < 0.05, respectively), and ammonia levels in the medium were significantly higher than in the untreated group (p < 0.01) (Figures 4D,E).

Immunocytochemical analyses corroborated these results (Figures 4F,G). Albumin and OTC were readily detectable in untreated samples but were nearly absent in tissues with acute damage, indicating compromised hepatocyte function. Conversely, fibrotic and HSC activation markers—including vimentin, α-SMA, and collagen-1—were negligible under untreated conditions yet strongly expressed in the acute model, underscoring a rapid and intense fibrogenic response. Lipid staining further showed minimal accumulation in the untreated samples, whereas the acute damage condition exhibited abundant, enlarged lipid droplets, revealing a severe disruption in lipid metabolism.

Together, these results highlight the profound impact of acute injury on hepatocyte function, metabolic homeostasis, and nuclear morphology, as well as the rapid activation of HSCs and fibrotic pathways. The elevated ammonia levels, decline in hepatocyte-specific markers, and rise in fibrotic markers support the utility of this 3D liver model for recapitulating key features of acute liver damage.

### Validation of the chronic and acute models using dexamethasone (100 nM)

**FIGURE 5:**
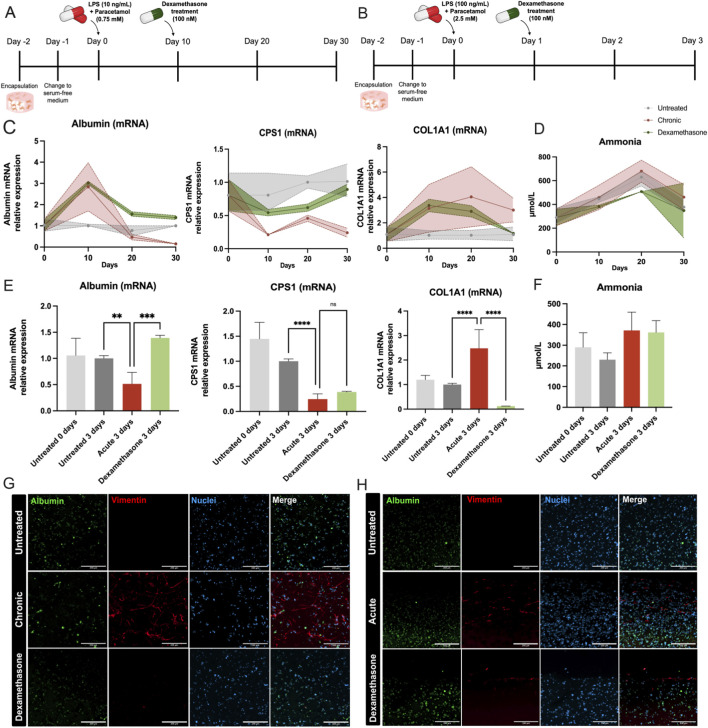
Effects of dexamethasone treatment on chronic and acute liver damage in the 3D liver model. **(A,B)** Experimental timelines for chronic **(A)** and acute **(B)** liver damage models. Encapsulation was performed on Day −2, followed by a switch to serum-free medium (SFM) on Day −1. For the chronic model, liver damage was induced with LPS (10 ng/mL) and Paracetamol (0.75 mM) on Day 0, and dexamethasone treatment (100 nM) began on Day 2. For the acute model, liver damage was induced with higher concentrations of LPS (100 ng/mL) and Paracetamol (2.5 mM) on Day 0, and dexamethasone was applied on Day 1. **(C,E)** mRNA expression of hepatocyte-specific markers (Albumin, CPS1) and the fibrotic marker Col1α1 in the chronic and acute damage model measured by qPCR. **(D–F)** Ammonia levels in chronic and acute damage models. **(G,H)** Immunofluorescence staining for Albumin (green), vimentin (red), and nuclei (blue) in chronic and acute models. The statistical significance is denoted as follows: *= 0.05; **= 0.01; ***= 0.001; ****= 0.0001. These results demonstrate the protective and anti-fibrotic effects of dexamethasone in both chronic and acute liver damage models, highlighting its ability to restore hepatocyte function, reduce fibrosis, and improve metabolic activity in the 3D liver model.

Dexamethasone, a potent synthetic glucocorticoid with anti-inflammatory, immunosuppressive, and anti-fibrotic properties, was validated in both chronic and acute 3D liver damage models. The experimental setup involved subjecting 3D liver tissues to LPS and Paracetamol at 10 ng/mL and 0.75 mM, respectively, for the chronic model and at 100 ng/mL and 2.5 mM for the acute model. After 1 day in growth medium (GM) and 2 days in serum-free medium (SFM) for differentiation, the liver tissues were exposed to the LPS + Paracetamol challenge. For the chronic model, analyses were conducted on days 0, 10, 20, and 30 following exposures, whereas the acute model was analyzed on day 3. Dexamethasone treatment commenced on day 10 for the chronic model and on day 1 for the acute model, both times post-challenge with LPS + Paracetamol (Figures 5A,B).

Gene expression profiles of albumin, CPS1, and Col1a1 were evaluated to determine the efficacy of dexamethasone. In the chronic model, mRNA levels of albumin and CPS1 rose significantly, approaching those in the untreated samples by day 30, while Col1a1 levels declined markedly. These changes indicate a restoration of hepatocyte function, accompanied by reduced fibrotic activity. In the acute model, albumin and Col1a1 returned to near-untreated levels, showing significant increases and decreases, respectively, although CPS1 expression remained unchanged (Figures 5E,F. Ammonia levels in the chronic model decreased under dexamethasone treatment, albeit with high variability; in contrast, dexamethasone did not improve ammonia levels in the acute model (Figures 5D,F).

Immunohistochemistry provided further corroboration. In both chronic and acute conditions, LPS + Paracetamol treatment significantly reduced the albumin signal and increased vimentin staining compared to untreated samples, indicating hepatocyte dysfunction and HSC activation. When dexamethasone was added, the albumin signal increased, while vimentin staining was attenuated, indicating the inactivation of HSCs (Figures 5G,H). These observations align with the gene expression data and underscore the protective, anti-fibrotic influence of dexamethasone in countering LPS + Paracetamol-induced damage.

#### Immune tri-culture system

Macrophages constitute a critical component of the innate immune system, playing an indispensable role in the inflammatory response. Under pathological conditions, additional macrophages from the systemic immune system are recruited to the liver, supplementing resident Kupffer cells. This influx drives disease progression by perpetuating inflammation and tissue injury. Given this key role of macrophages, we sought to incorporate them into our 3D liver model to investigate how soluble factors released by healthy or diseased liver tissues influence monocyte behavior.

#### Tri-culture setup and experimental design

**FIGURE 6:**
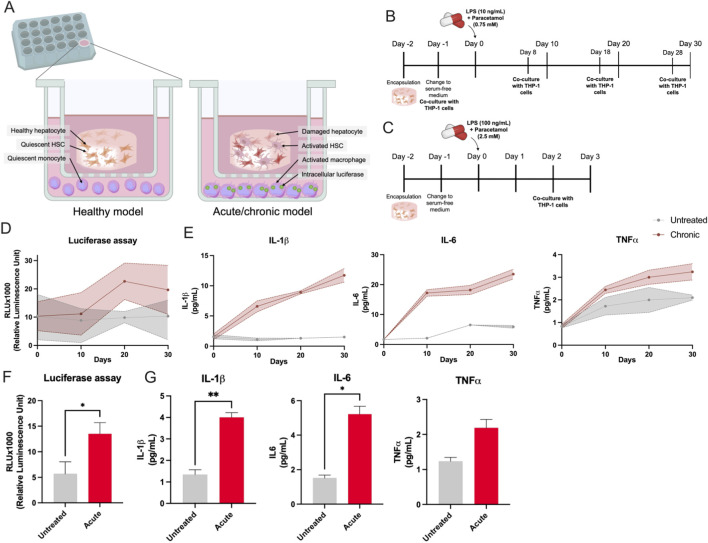
Inflammatory response in the chronic and acute damage models co-cultured with THP-1 NF-κB-Luc2 cells. **(A)** Schematic representation of healthy and acute/chronic liver damage models in co-culture with THP-1 NF-κB-Luc2 reporter cells. **(B,C)** Experimental timelines. In the chronic model **(B)**, LPS (10 ng/mL) and Paracetamol (0.75 mM) were added on Day 0, followed by co-culture with THP-1 NF-κB-Luc2 reporter cells starting on Day −1 and continuing through Days 8, 18, and 28. In the acute model **(C)**, higher concentrations of LPS (100 ng/mL) and Paracetamol (2.5 mM) were applied on Day 0, and THP-1 NF-κB-Luc2 reporter cells co-culture was initiated on Day 2 and analyzed on Day 3. **(D,F)** Luciferase assay in the chronic and acute models. **(E,G)** Levels of pro-inflammatory cytokines (IL-6, IL-1β, and TNFα) in the chronic, up to 30 days, and acute models, up to 3 days, were measured by Luminex assay in the medium. These results demonstrate the activation of the NF-κB pathway and associated inflammatory cytokines in both chronic and acute damage models. The statistical significance is denoted as follows: *= 0.05; **= 0.01; ***= 0.001; ****= 0.0001. The chronic model exhibits sustained inflammation over time, while the acute model shows a rapid and intense inflammatory response. This highlights the utility of the 3D liver model in studying inflammatory signaling in liver injury.

To achieve this goal, we developed a tri-culture system comprising engineered 3D liver tissues in both acute and chronic damage states, along with monocytes (Figure 6A). The 3D liver tissues were prepared and maintained under the previously described acute and chronic conditions, then placed in the upper compartment of transwell inserts. Meanwhile, monocytes were cultured in suspension in 24-well plates situated below each insert. The semi-permeable membrane allowed soluble factors to diffuse between compartments without direct cell-cell contact. In the acute damage model, monocytes were introduced into the co-culture on Day 2 of LPS-Paracetamol treatment, and immune cells were assessed on Day 3 (Figure 6C). For the chronic damage model (Figure 6B), monocytes were added on Days −1, 8, 18, and 28 of treatment, and samples were collected and analyzed on Days 0, 10, 20, and 30. These time points were selected to capture distinct phases of liver tissue injury and recovery.

### Activation of NF-κB pathway under chronic and acute liver damage conditions

The results from the luciferase assay, using THP-1 NF-κB-Luc2 (TIB-202-NFkB-LUC2™) reporter monocytes, highlight the activation of the NF-κB pathway under both chronic and acute damage conditions. The chronic damage model exhibits a time-dependent increase in NF-κB activity, as indicated by the rising luciferase levels (RLU x 1,000) from Days 10–30. In contrast, NF-κB activity remains relatively stable and significantly lower in untreated conditions across time points (Figure 6D). In the acute damage model, NF-κB activity is significantly elevated (p < 0.05) compared to the untreated condition, suggesting rapid activation of inflammatory signaling pathways. These results indicate that both acute and chronic damage conditions robustly activate the NF-κB pathway in THP1-Lucia™ NF-κB cells, reflecting the inflammatory response induced by these treatments. This aligns with the expected role of NF-κB as a key mediator of inflammation in response to liver damage (Figure 6F).

### Cytokine release and NF-κB activation

To evaluate the inflammatory response, we collected the culture supernatants from the co-cultures to measure the release of key pro-inflammatory cytokines—interleukin-6 (IL-6), interleukin-1β (IL-1β), and tumor necrosis factor-α (TNF-α)—by activated macrophages. No additional LPS-Paracetamol was introduced during this evaluation; instead, fresh medium was used to avoid confounding effects of exogenous stimulation. All three cytokines displayed similar overall trends, with significantly elevated levels in acute and chronic models compared to untreated controls.

In the chronic model, cytokine concentrations steadily rose at Days 10, 20, and 30, correlating with ongoing tissue injury (Figure 6E). The acute model also showed higher cytokine levels than untreated samples, reflecting a rapid immune response to short-term damage (Figure 6G).

Monocyte activation was further monitored using THP-1 cells harboring an NF-κB-driven luciferase reporter, which enabled quantitative luminescence measurements. Both the acute and chronic damage models exhibited substantial increases in luminescence relative to the untreated state, indicating heightened NF-κB activity and a robust inflammatory response.

Including macrophages in our 3D liver model is valuable for examining immune-mediated processes underlying liver injury. This tri-culture system highlights how acute and chronic liver damage forms can differentially modulate macrophage activation through soluble factors. Elevated cytokine production and NF-κB activation in co-cultures underscore the significance of macrophages in driving and perpetuating liver pathology, offering a relevant platform for future therapeutic intervention studies.

## Discussion

In this study, we present a physiologically relevant three-dimensional (3D) human liver model that successfully recreates both acute and chronic drug-induced liver injury (DILI) (Figure 1A). While several cellular models have been established to investigate acute hepatotoxicity, evaluating chronic liver damage has traditionally relied on animal models (Smith, 2024; Ma, 2009). Two-dimensional (2D) cultures lose key liver functions over time and fail to capture the sustained, low-dose injury characteristic of chronic conditions (Guo K, 2024; Serras AS, 2021). Our 3D model addresses this gap by maintaining liver-specific functions for up to 30 days, thus enabling the study of long-term hepatotoxicity. Additionally, it incorporates an immune tri-culture system, allowing us to explore the role of macrophages in liver pathology. These findings underscore the importance of 3D systems in revealing mechanisms of cumulative and delayed toxicity that are typically overlooked in conventional 2D models.

Liver tissue engineering requires encapsulating hepatic cells within scaffolds that provide appropriate porosity, biochemical cues, topography, and stiffness. In this study, we aimed to fabricate long-lasting engineered liver tissues. GelMA hydrogels, derived from gelatin, were selected for their cell-adhesion motifs and photocrosslinkable properties. To improve stability, GelMA was copolymerized with CMCMA, a polymer resistant to enzymatic degradation. The optimization and characterization of GelMA-CMCMA hydrogels were described in our previous work (García-Lizarribar et al., 2018).

The resulting 3D GelMA-CMCMA hydrogels provided a stable, physiologically relevant microenvironment, supporting hepatocyte function and maintaining hepatic stellate cell (HSC) quiescence. With stiffness comparable to healthy liver (≈1.5–4.5 kPa), the model outperforms conventional 2D cultures. Co-cultures of HepaRG and LX-2 cells under serum-free conditions for 30 days successfully recapitulated key features of the hepatic niche, enabling the study of chronic processes.

The hydrogels allowed cells to form multicellular clusters and maintain liver-specific functions over time. Ring-shaped molds were used to produce mechanically stable scaffolds that promote cellular self-organization. While this geometry does not replicate the hepatic lobule, it supports physiologically relevant cell-cell and cell-matrix interactions: hepatocytes remained functional, HSCs stayed quiescent, and monocytes in indirect contact were not activated under basal conditions.

One of the principal achievements of this work is the model’s capacity to support extended cell viability and phenotypic maintenance for up to 30 days, as indicated by sustained albumin expression, stable cytochrome P450 (CYP450) activity, and effective ammonia detoxification pathways in untreated samples (Zurlo J, 1996; Bachour-El Azzi, 2022; Huch M, 2015) (Figures 1C,D,G). The inclusion of hepatic stellate cells (HSCs) in the 3D GelMA matrix prevented their spontaneous activation, a frequent complication when HSCs are cultured on rigid 2D substrates (Brovold, 2021; Brovold, 2020). This quiescent state of HSCs is essential for accurately studying fibrogenic processes upon injury. Over the 30-day culture, the HSCs remained inactive under untreated conditions yet became activated upon exposure to chronic or acute insults, reinforcing the physiological relevance of the model for investigating liver fibrosis (Figures 1E,G). To rule out the possibility that the late-stage decline in activation markers was due to stellate-cell loss rather than true de-activation, we examined viability and injury-response data. Live/Dead assays indicated >90% total viability at every time point, suggesting no selective loss of LX-2 cells. Notably, under both acute and chronic insults, stellate-cell activation markers, extracellular collagen, and PIIINP/PINP secretion increased, with cells exhibiting α-SMA-positive morphology as shown by confocal microscopy. These contrasting trends—downward in untreated cultures and upward under injury—demonstrate that stellate cells persist and revert to quiescence within the GelMA-CMCMA matrix instead of being lost.

Our results highlight the importance of cell clustering within the 3D matrix (Bell CC, 2016; Nagata S, 2020). By Day 10, hepatocytes formed clusters that demonstrated more robust albumin expression compared to isolated cells, suggesting that these 3D configurations facilitate cell-cell interactions necessary for high-level hepatic function (Figure 1H). This phenomenon provides a plausible explanation for the increased functionality we observed in the 3D system: cell-cell and cell-matrix contacts more closely replicate *in vivo* conditions than monolayer cultures, which typically lack the spatial cues required to sustain hepatic differentiation.

Distinct molecular and functional analyses confirmed the model’s capability to reflect both acute and chronic liver damage. Long-term exposure to low-dose LPS and Paracetamol caused sustained hepatocyte injury and inflammation, leading to significant transcriptional changes by Days 10, 20, and 30. Gene set enrichments indicated increased fibrotic and HSC activation pathways, along with decreased hepatocyte function. Confocal imaging showed progressive collagen I deposition, vimentin upregulation, and lipid accumulation, illustrating the relationship between fibrosis and steatosis under chronic stress. RNA-seq revealed disturbances in fatty acid biosynthesis/oxidation and ceramide accumulation, indicating metabolic reprogramming.

Lipid droplets accumulated in the chronic model despite being cultured in serum-free medium without added fats. RNA-seq data revealed dysregulation in fatty acid metabolism: upstream enzymes like ACACA and ACLY were upregulated, while downstream enzymes were suppressed, inhibiting fatty acid elongation and oxidation. This disruption likely caused lipid accumulation in hepatocytes, worsened by abnormal ceramide metabolism, explaining the persistence of steatosis without extracellular fatty acids.

Ammonia levels in untreated samples fluctuated without a direct correlation to urea cycle enzyme mRNA levels like CPS1 and OTC, highlighting the complex regulation of ammonia detoxification. In contrast, the chronic damage model showed a clear disruption in the urea cycle, marked by reduced expression of Albumin, CPS1 and OTC (Nielsen SS, 2007; Eriksen, 2019). This diminished detoxification capacity aligns with known features of chronic liver disease, emphasizing the model’s utility in reproducing pathophysiological conditions over extended timeframes. RT-qPCR showed biphasic Albumin and OTC expression, with early upregulation at Day 10 followed by a sustained decline. The initial rise reflects a transient compensatory response, consistent with increased hepatocyte activity during early regeneration. Urea cycle enzymes like OTC support ammonia clearance, but their later decrease likely marks exhaustion of this reserve and progression toward chronic hepatocyte dysfunction, mirroring hallmarks of advanced liver injury such as impaired urea cycle function and reduced albumin synthesis (Barle et al., 2006; Amouzandeh et al., 2023; Rutherford and Chung, 2008).

The GelMA-CMCMA scaffold presents pore sizes ranging from 200–600 nm. This nanoscale tortuosity and polymer adsorption slow small-molecule diffusion. Consequently, paracetamol reaches hepatocytes far more gradually than in a 2D monolayer, tempering the rapid necrosis typically observed in flat-plate cultures. The time-course profiles in Figure 4B confirm this intended buffering effect. By applying higher concentrations of LPS and Paracetamol, we reproduced acute hepatic damage characterized by sharp declines in albumin and CPS1 expression, reduced CYP3A4 activity, and elevated ammonia levels (Figures 4C–E). Confocal microscopy confirmed rapid HSC activation (e.g., increased α-SMA, collagen-1) and compromised hepatocyte integrity (Figure 4G). These hallmarks mimic scenarios of acute liver failure, validating the model’s flexibility in capturing different injury kinetics.

We selected dexamethasone—rather than the antioxidant N-acetylcysteine (NAC)—as our benchmark anti-injury agent because its dual actions preserve hepatocyte metabolic function and suppress THP-1–driven inflammatory signaling, thereby providing a more comprehensive test of therapeutic rescue in this multicellular 3D liver model. In contrast, NAC’s efficacy is largely confined to the oxidative-stress axis of Paracetamol toxicity and offers limited immunomodulatory Benefit (Tasnim et al., 2021; Bhandari et al., 2024). Dexamethasone successfully mitigated both chronic and acute injury states, reverting albumin and CPS1 expression to untreated levels while lowering fibrogenic markers, such as Col1α1 (Figure 5C). Immunohistochemistry (Figure 5G) corroborated these improvements, showing reduced vimentin staining and increased albumin signal (Imoto K, 2022). This data confirms dexamethasone’s well-established anti-inflammatory and antifibrotic effects, underscoring the potential of our system for preclinical testing of therapeutic agents.

In addition to studying the responses of hepatocytes and HSCs, we incorporated monocytes (THP-1 cells) to form an immune tri-culture. This enabled us to observe the interplay between immune cells and liver parenchymal and non-parenchymal cells. The integration of THP-1 monocytes in a transwell co-culture format revealed their potent response to both acute and chronic injury, as evidenced by NF-κB–driven luciferase activity and cytokine release (Figures 6D–G). IL-6, IL-1β, and TNF-α increased significantly under damage conditions, mirroring the inflammation typically observed *in vivo*. Macrophage and monocyte recruitment is vital in the pathogenesis of liver disease, as these cells secrete inflammatory mediators that aggravate hepatocyte injury and promote further HSC activation (Gröger M, 2016). These findings emphasize the critical role of macrophages in liver injury and the need to integrate immune components into advanced liver models.

However, our 3D liver model has limitations. It does not fully represent other non-parenchymal liver cells (like endothelial cells and Kupffer cells), which may affect its ability to capture the full complexity of liver pathophysiology. Additionally, patient-specific genetic and etiological factors are not accounted for when using standardized cell lines. While there is a strong correlation between gene expression and functional outcomes, further enzymatic assays and proteomic analyses are needed to clarify the post-translational mechanisms involved in hepatic function.

Increasing the cellular complexity of the model—either by incorporating additional liver cell types or by using primary human cells or induced pluripotent stem cell (iPSC)-derived hepatocytes—could further enhance its physiological relevance. Moreover, optimizing the hydrogel matrix’s stiffness or composition may allow for more nuanced control of HSC activation and endothelial cell function. Finally, coupling this model with organ-on-a-chip platforms or perfusion bioreactors could more closely mimic hepatic blood flow dynamics, thereby enabling real-time analyses of drug metabolism and clearance.

Overall, these findings highlight the utility of our 3D liver model in capturing essential facets of liver pathophysiology under both chronic and acute challenges. By combining parenchymal and non-parenchymal cell populations, along with an immune tri-culture system, the model recapitulates complex injury responses—fibrogenesis, metabolic shifts, and pro-inflammatory signaling—as 2D cultures typically lack spatial and immunological complexity. Importantly, the consistent success of dexamethasone treatment in restoring homeostasis underscores the model’s potential as a preclinical tool for evaluating novel therapeutics. Looking ahead, continued refinements and increased cellular complexity (e.g., cholangiocytes, endothelial cells) will likely further enhance the model’s translational impact, providing a powerful platform for understanding liver disease progression and accelerating the development of effective treatments.

## Materials and methods

### Cell culture

HepaRG is a human hepatic cell line that preserves many characteristics of primary human hepatocytes. The cells were cultured and expanded in William’s medium at 37 °C in 5% CO_2_. William’s medium was supplemented with 10% fetal bovine serum (FBS), insulin, hydrocortisone succinate, and 1% penicillin-streptomycin (growth medium). For differentiation, the cells were cultured in William’s medium and serum-free medium supplement (HPRG750, Thermo Fisher Scientific). All the experiments were conducted in serum-free medium conditions. The detailed composition of the supplement is proprietary and is not disclosed by the manufacturer.

LX-2 is a human hepatic stellate cell line. The cells were cultured and amplified in Dulbecco’s modified Eagle medium (DMEM) at 37 °C in 5% CO_2_. DMEM was supplemented with 2% FBS and 1% penicillin-streptomycin. For cell subculture, trypsin-EDTA (25200072, ThermoFisher, Washington, DC, United States of America) and phosphate-buffered saline (PBS) were used.

THP-1 NF-κB-Luc2 (TIB-202-NFkB-LUC2™) is a monocyte human cell line widely utilized to study monocyte and macrophage functions. This cell line stably expresses the firefly luciferase gene (luc2) under the control of an NF-kB promoter. Upon stimulation, the cells express high levels of enzymatically active luciferase protein, detectable via *in vitro* bioluminescence assays. This reporter cell line is valuable for monitoring inflammation levels in an *in vitro* system. The cells were cultured and expanded in RPMI medium at 37 °C in 5% CO_2_. RPMI medium was supplemented with 10% FBS and 1% penicillin-streptomycin, 2-mercaptoethanol 0.05 mM and puromycin 1 μg/mL.

#### Cell encapsulation

The hydrogel utilized for liver tissues has been detailed in a previous publication (De Chiara F, 2022). Briefly, the hydrogel comprised a blend of gelatin methacryloyl (GelMA, 5% w/v) and carboxymethyl cellulose methacrylate (CMCMA, 1% w/v), representing biodegradable and non-biodegradable materials, respectively (e.g., for preparing 500 µL of the prepolymer solution, 0.05 g of GelMA and 0.01 g of CMCMA were weighed). The mixture was subjected to sterilization with UV for 15 min and then incubated in 400 µL of culture medium at 65 °C for 3 h. After complete pre-polymer dissolution, lithium phenyl(2,4,6-trimethylbenzoyl) phosphonate (LAP, 0.1% w/v) was added to the GelMA-CMCMA mixture as a photo-initiator in culture media. Subsequently, cells (immortalized human hepatocytes and HSCs in a 2:1 ratio) were combined with the GelMA-CMCMA mixture in a 1:1 ratio and exposed to UV for 30 s. Henceforth, the pre-polymer and cell mixture will be denoted as liver tissue. The liver tissues had a final volume of 50 μL, with a cell concentration of 1.5 × 10^4^ cells/µL. To impart a specific shape to the liver tissues, polydimethylsiloxane (PDMS) moulds were employed (Figure 1A). Ring-shaped moulds with an inner diameter of 5 mm and an outer diameter of 8 mm were used for cell encapsulation. The hydrogel fabrication plate used was a standard 24-well plate. After encapsulation, the liver tissues were cultured with growth medium for 1 day and then with differentiation medium, with fresh medium replaced every 2–3 days. Liver tissues were maintained in culture for 30 days. Importantly, the 30-s exposure used here does not introduce biologically relevant DNA damage. Polymerisation was performed with 365 nm UV-A at 3 mW cm^-2^ for 30 s with a Fluence of 0.09 J/cm2 (Fluence = Exposure time × Irradiance). According to ISO-standard photobiology safety limits for mammalian cells, the threshold fluence for Cyclobutane pyrimidine dimer formation, exceeding repair mechanisms, is greater than 1.27 J/cm^2^. Fluences above 2.54 J/cm^2^ are predicted to inhibit repair mechanisms, leading to an accumulation of DNA damage.

#### Tri-Culture System

We established a tri-culture system consisting of liver tissues and monocytes to investigate immune system interactions. Liver tissues were fabricated and cultured under previously described conditions of acute and chronic damage. For tri-culture experiments, the liver tissues were placed in the upper compartment of a transwell insert, while monocytes were cultured in suspension in the lower wells of a 24-well plate. The 2 cell types were separated by a semi-permeable membrane, allowing paracrine interactions during the tri-culture period (24–48 h). For the acute damage model, liver tissues and monocytes were co-cultured on day 2 of treatment, and immune cell analysis was performed on day 3. For the chronic damage model, co-culture was conducted on days −1, 8, 18, and 28 of treatment, with immune cell analysis carried out on days 0, 10, 20, and 30. The culture medium supernatant was collected from the co-cultures at the end of the specified incubation periods for downstream analysis.

#### Drug treatments

To induce the chronic liver damage, the liver tissues were challenged at day 0 (48 h after encapsulation) with Paracetamol 0.75 mM and LPS 10 ng/mL in SFM for 30 days (replenished every medium change). To induce the acute liver damage, the liver tissues were challenged at day 0 (48 h after encapsulation) with Paracetamol 2.5 mM and LPS 100 ng/mL in SFM for 3 days (single administration).

To validate the acute and chronic damage liver models with an anti-fibrotic and anti-inflammatory drug, the liver tissues were treated with 100 nM dexamethasone in SFM. A stock solution of 10 mM dexamethasone in absolute ethanol was prepared and diluted with SFM to make a final concentration of 100 nM.

#### CellTiter 96® aqueous one solution cell proliferation assay (MTS)

To assess cell viability, MTS (G3582, Walldorf, Germany) was introduced into the wells where the cells were previously seeded, maintaining a media to MTS ratio of 1:5. For instance, in a 96-well plate, 20 µL of MTS was added to 100 µL/well of fresh media and incubated at 37 °C for 2 h in a humidified, 5% CO_2_ atmosphere. The absorbance at 490 nm was then employed to quantify the amount of soluble formazan produced through the cellular reduction of MTS.

#### AdipoRed™ assay

To quantify intracellular triglyceride accumulation, AdipoRed™ (PT-7009, Lonza, WL, Germany) solution was utilized (Supplementary Table S1). The volume employed varied based on the plate size. For instance, in 96-well plates, the AdipoRed™ volume per well was 5 µL in 195 µL of phenol red-free media. Following each experiment, the plates were taken out of the incubator and assayed for intracellular triglyceride content. Cells were rinsed twice with 200 µL of PBS to eliminate any residual media. Incubation times were 10 and 30 min for 2D and 3D experimental setups, respectively. Subsequently, fluorescence was measured using a fluorimeter with excitation at 485 nm and emission at 572 nm, and images were captured using an inverted microscope.

### Live/Dead staining

For each test, ethidium homodimer-1 (2 µM) (L3224, ThermoFisher, Washington, DC, United States of America) and calcein-AM (4 µM) (Supplementary Table S1) were combined with PBS following the manufacturer’s protocol. Subsequently, the plate was incubated for 30 min and washed with PBS three times. The hydrogels were then examined under confocal microscopy. For longitudinal quantification, we computed the calcein-positive area per image stack with identical exposure settings and normalised values to hydrogel volume.

### Immunofluorescence staining

Samples were fixed in a 10% formalin solution, rinsed with PBS, and permeabilized with Triton X-100 at 0.1% in PBS for 10 min. Subsequently, samples were subjected to overnight incubation with various antibodies, including albumin antibody, OTC antibody, vimentin, alpha-SMA, and collagen-I antibody (Supplementary Table S2), in PBS at 4 °C. The following day, excess antibodies were washed off with PBS, and the samples were then incubated with rhodamine-phalloidin and different secondary antibodies, such as goat anti-rabbit and goat anti-mouse (Supplementary Table S3), in a blocking solution for 2 h. Nuclei were stained with 1 µM DAPI for 30 min (Supplementary Table S1).

### Imaging and quantitative morphological analysis

Liver tissues were analysed using confocal imaging with ZEISS LSM800 microscope. 5x, 10x, ×20 and ×40 magnifications were used to procure images from the confocal microscope. After the acquisition, images were processed and analysed using Fiji/ImageJ. For the Live/Dead assay, the number of live and dead cells was quantified by counting fluorescent signals corresponding to live (green fluorescence) and dead (red fluorescence) cells. Cell counts were obtained using ImageJ’s “Cell Counter” plugin, ensuring consistent thresholding across all images. Nuclei were identified based on their fluorescence for the nuclear size quantification, and their sizes were measured using the “Analyse Particles” function in ImageJ. Images were pre-processed by adjusting the brightness and contrast, and a consistent threshold was applied to ensure accurate segmentation of nuclear regions. The average nuclear area was calculated from at least three independent images per condition.

#### P450 cytochrome assay

Vivid® CYP450 Screening Kit was used to assess the metabolism and inhibition of the P450 isozyme CYP3A4, which is involved in hepatic drug metabolism.

#### Real-time qPCR

Liver tissues were homogenized in QIAzol reagent (QIAGEN) using sterilized pestles, and total RNA was isolated with the miRNeasy Micro Kit (QIAGEN) following the manufacturer’s protocol. RNA samples (1 μg) were treated with DNase I (Invitrogen) to remove genomic DNA and subsequently reverse transcribed into cDNA using SuperScript II (Invitrogen) with random hexamer primers. Quantitative real-time PCR (qRT-PCR) was performed in triplicate for each biological replicate using 10 ng of cDNA and TaqMan probes (Supplementary Table S4). Actin beta was used as the endogenous control. Thermal cycling was conducted on the StepOne Plus RT-PCR System (Applied Biosystems). Relative gene expression compared to the control group and normalized to the endogenous gene was calculated using the 2^−ΔΔCT^ method.

#### Luciferase assay

Pierce Firefly Luciferase Flash Assay kit (16,175, Thermo Fisher Scientific, MA, United States of America) was used to detect intracellular luciferase activity from the activated THP-1 cells. The bioluminescent signal produced by the firefly luciferase is proportional to the activity of the promoter for firefly expression.

#### Cytokines analysis

According to the manufacturer’s instructions, the levels of IL-6, IL-1ß and TNF-alpha were determined using the ProcartaPlex™ Human MMP Panel 2, 3plex (Invitrogen ™, Thermo Fisher Scientific, MA, United States of America). The plates were read using the MAGPIX magnetic bead reader (LuminexCorp., Austin, TX, United States of America) based on the xPONENT® 4.2 software (Luminex).

### RNA sequencing

#### Sample preparation

3D liver tissues were harvested, flash-frozen in liquid nitrogen, and stored at −80 °C until RNA extraction. For stabilization, samples could also be preserved in RNAlater® (Thermo Fisher Scientific) and stored as per the manufacturer’s recommendations.

#### RNA extraction

Total RNA was extracted from tissues using the Qiagen RNeasy Mini Kit following the manufacturer’s instructions. Tissue homogenization was performed using a bead homogenizer. Genomic DNA contamination was removed by treating RNA samples with DNase I (Qiagen). RNA concentration and purity were assessed using a NanoDrop spectrophotometer, and RNA integrity was evaluated using an Agilent 2,100 Bioanalyzer. Only samples with an RNA Integrity Number (RIN) ≥ 7 were used for further analysis.

#### mRNA enrichment

Polyadenylated RNA was enriched from total RNA using the NEBNext Poly(A) mRNA Magnetic Isolation Module (New England Biolabs). For total RNA workflows, rRNA was depleted using the NEBNext rRNA Depletion Kit.

#### cDNA synthesis

Enriched mRNA was reverse-transcribed into first-strand cDNA using the NEBNext First Strand Synthesis Module, followed by second-strand synthesis to produce double-stranded cDNA.

#### Library preparation

Double-stranded cDNA was fragmented to an average size of 200 bp using the NEBNext Ultra II Directional RNA Library Prep Kit for Illumina. Fragmented cDNA underwent end-repair, A-tailing, and adapter ligation. Libraries were amplified with indexing primers through PCR and purified using AMPure XP beads (Beckman Coulter). The quality and concentration of libraries were assessed using an Agilent Bioanalyzer.

#### Sequencing

Final libraries were pooled at equimolar concentrations and sequenced on the Illumina NovaSeq platform with paired-end 100 bp reads. Sequencing depth was designed to achieve at least 30 million reads per sample.

#### Data analysis

Raw sequencing reads were subjected to quality control using FastQC. Low-quality reads and adapter sequences were trimmed using Trimmomatic. Reads were aligned to the human reference genome (GRCh38) using the STAR aligner. Gene expression quantification was performed with featureCounts, and differential expression analysis was conducted using DESeq2. Genes with a false discovery rate (FDR) <0.05 were considered significantly differentially expressed. Pathway enrichment analysis was performed using the Gene Set Enrichment Analysis (GSEA) tool.

## Measurement of ammonia, PINP, and PIIINP

Ammonia levels were quantified using an enzymatic colorimetric assay on an automated chemistry analyzer (Roche Cobas). PINP (Procollagen Type I N-terminal Propeptide) levels were measured using an automated immunoassay analyzer (IDS-iSYS). PIIINP (Procollagen Type III N-terminal Propeptide) levels were quantified using a specific ELISA. Standards, controls, and samples were incubated in antibody-coated wells, followed by signal development and absorbance measurement at 450 nm. All assays included internal quality controls and calibrators provided by the manufacturers. Runs were repeated if control values fell outside the acceptable range to ensure accuracy and reliability of the measurements.

## Statistics

All experiments were performed in biological triplicates-quintuplets with at least two technical replicates per condition. Results are expressed as mean values SEM and compared using one-way analysis of variance followed by Dunnet’s or Tukey’s multiple comparison *post hoc* tests, where appropriate. p values 0.05 were considered significant. Results were analyzed using GraphPad Prism software.

## Data Availability

The original contributions presented in the study are publicly available. This data can be found here: CORA.Repositori de Dades de Recerca, https://doi.org/10.34810/data27.
