# Supplementary material for: A 3D bioengineered human liver for the study of acute and chronic drug-induced hepatotoxicity and fibrosis
Source: Front Bioeng Biotechnol. 2026 May 11;14:1798323. doi: 10.3389/fbioe.2026.1798323 (PMC13198925; doi:10.3389/fbioe.2026.1798323)
Supplement: Supplementary file 1 [file Supplementaryfile1.docx]

**Supplementary Material**

**A 3D Bioengineered Human Liver for the Study of Acute and Chronic Drug-Induced Hepatotoxicity and Fibrosis**

Supplementary Table 1. List of fluorescent stains for fluorescent staining

| Name | Working dilution | Catalog number | Company |
| --- | --- | --- | --- |
| DAPI | 1:1000 | D1306 | Life technologies (Thermo Fisher) |
| AdipoRedTM Assay Reagent | 1:40 | PT-7009 | Lonza |
| Hoechst | 1:1000 | 62249 | Life technologies (Thermo Fisher) |
| LIVE/DEAD™ Viability/Cytotoxicity Kit | 1:2000 | L3224 | Life technologies (Thermo Fisher) |

Supplementary Table 2. List of secondary antibodies for immunostaining

| Name | Host | Reactivity | Working dilution | Catalog number | Company |
| --- | --- | --- | --- | --- | --- |
| Albumin | Rabbit | Human, Mouse, Rat | 1:100 | PA5-85166 | Invitrogen |
| OTC | Rabbit | Human, Mouse, Rat | 1:200 | GTX64710 | GeneTex |
| Collagen-I | Mouse | Human | 1:200 | C2456 | Sigma |
| Vimentin | Mouse | Human | 1:200 | V2258 | Sigma |
| Alpha-SMA | Mouse | Human | 1:200 | D4K9N | Cell Signalling |

Supplementary Table 3. List of primary antibodies for immunostaining

| Name | Host | Reactivity | Working dilution | Catalog number | Company |
| --- | --- | --- | --- | --- | --- |
| Alexa Fluor 488 | Goat | Rabbit | 1:100 | A11034 | Invitrogen |
| Alexa Fluor plus 647 | Goat | Mouse | 1:200 | A32728 | Invitrogen |

Supplementary Table 4. List of genes for gene expression analysis

| Gene name | TaqMan™ Gene Expression Assay (FAM) ID |
| --- | --- |
| ALB | Hs00609411_m1 |
| OTC | Hs00166892_m1 |
| CPS1 | Hs00157048_m1 |
| COL1A1 | Hs00164004_m1 |
| ACTB | Hs01060665_g1 |

***Supplementary Figure S1. CYP3A4 enzymatic activity in 3D liver models measured at days 0, 10, 20, and 30 of culture.***

***Supplementary Figure S2. Brightfield images of 2D HepaRG cells and 3D liver tissues across days 10, 20 and 30.***


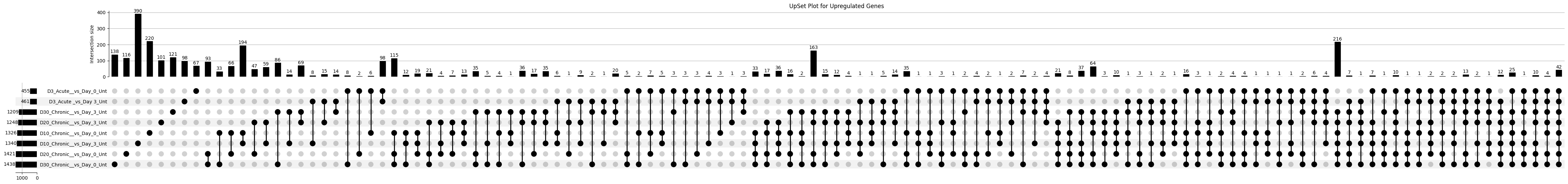


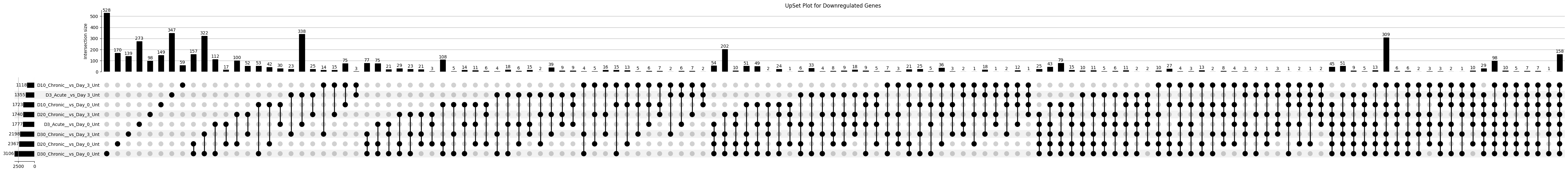


***Supplementary Figure S3. Differential gene expression across acute and chronic liver damage conditions.*** *Upset plots show the overlap and exclusivity of significantly upregulated (top) and downregulated (bottom) genes across acute and chronic liver damage conditions. Each vertical bar indicates the number of genes shared by the combination of conditions represented by the connected black dots below.*

Chronic Day 10 vs Untreated Day 10


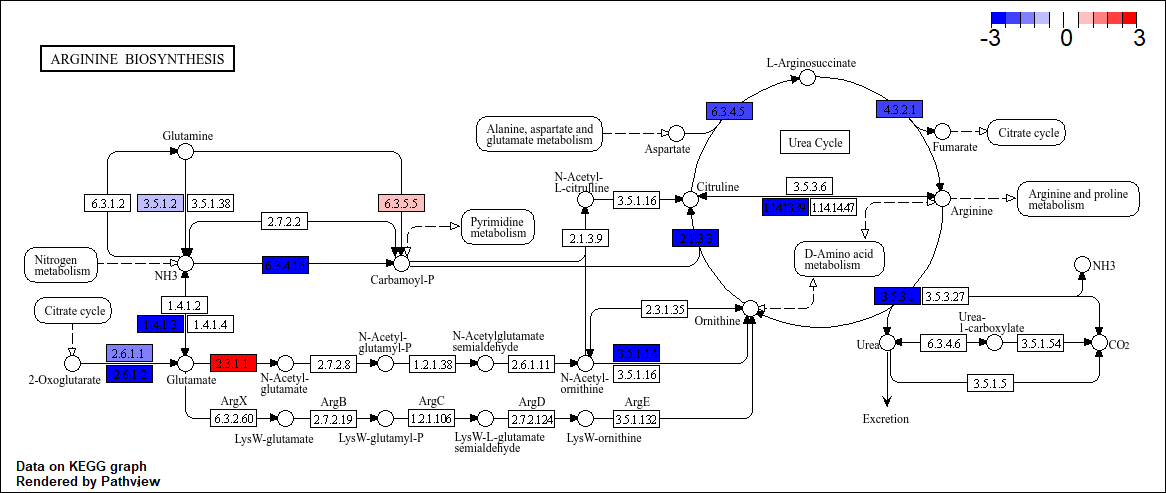

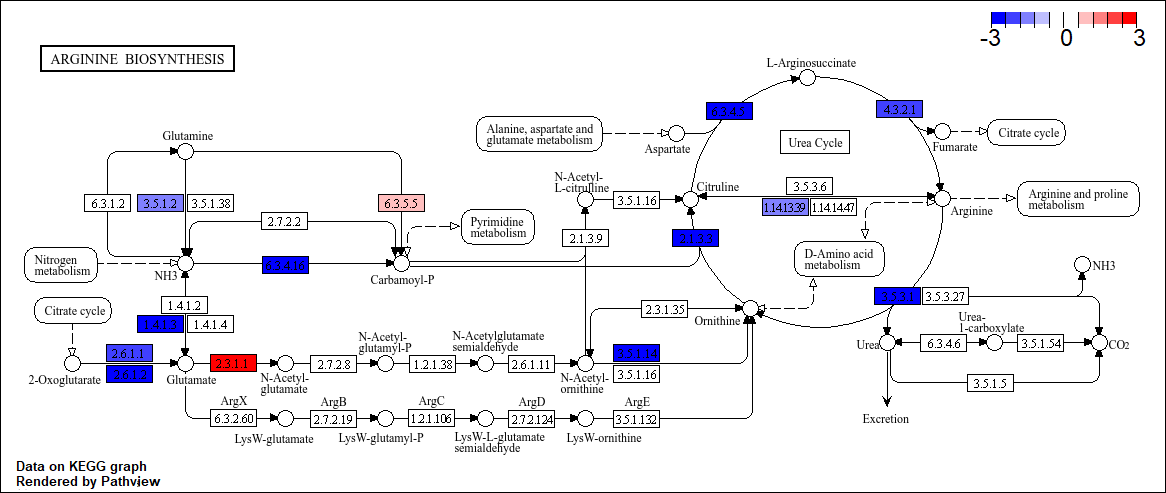


Chronic Day 20 vs Untreated Day 20


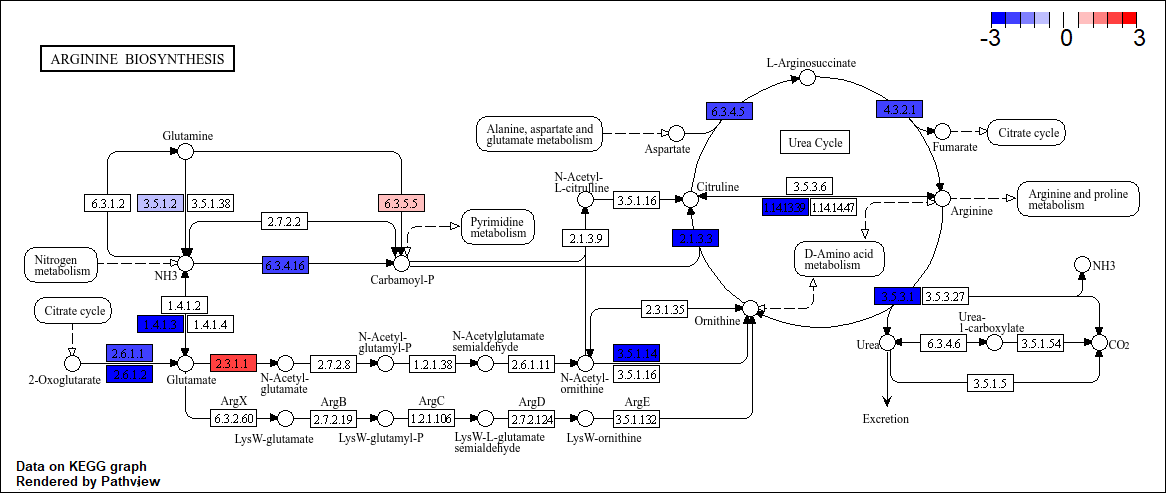


Chronic Day 30 vs Untreated Day 30


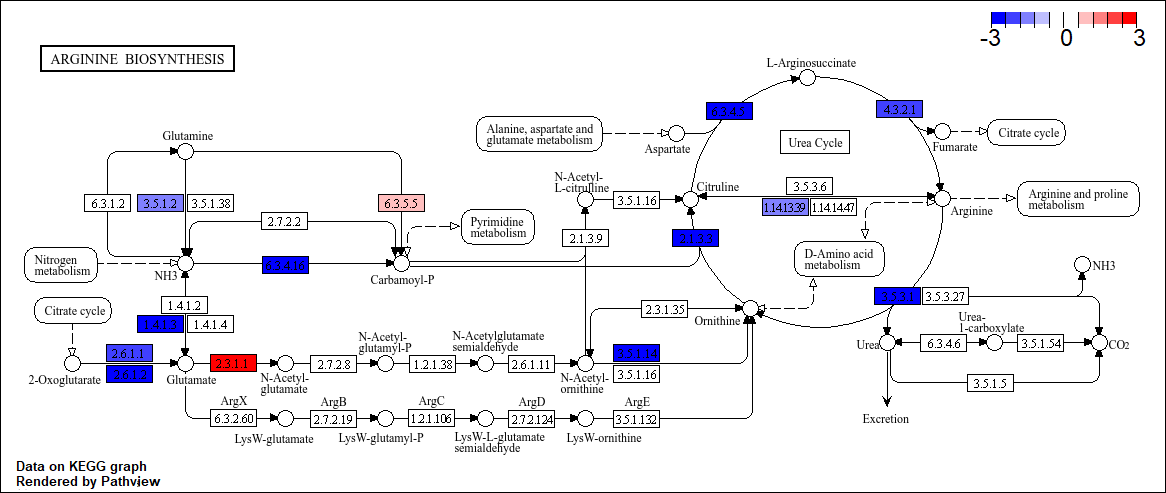


***Supplementary Figure S4. Pathway-level visualization of urea cycle gene expression in chronic damage versus untreated conditions across Days 10, 20, and 30.*** *Genes shown in blue are significantly downregulated, while those in red are significantly upregulated (adjusted p < 0.05).*

***Supplementary Figure S5. Protein levels of IL-6 and MCP-1 in 3D liver models at days 20 and 30 under untreated (healthy) and chronic liver damage conditions.***

***Supplementary Figure S6. Brightfield images of the 3D liver tissues comparing untreated and chronic damage conditions across days 10, 20 and 30.***
